# High-Loading-Dose Colistin with Nebulized Administration for Carbapenem-Resistant *Acinetobacter baumannii* Pneumonia in Critically Ill Patients: A Retrospective Cohort Study

**DOI:** 10.3390/antibiotics13030287

**Published:** 2024-03-21

**Authors:** Wasan Katip, Ajaree Rayanakorn, Chuleegone Sornsuvit, Purida Wientong, Peninnah Oberdorfer, Puntapong Taruangsri, Teerapong Nampuan

**Affiliations:** 1 Department of Pharmaceutical Care, Faculty of Pharmacy, Chiang Mai University, Chiang Mai 50200, Thailand; chuleegone.sornsuvit@cmu.ac.th (C.S.); purida.v@cmu.ac.th (P.W.); 2 Epidemiological and Innovative Research Group of Infectious Diseases (EIRGID), Faculty of Pharmacy, Chiang Mai University, Chiang Mai 50200, Thailand; ajaree.rayanakorn@cmu.ac.th (A.R.); peninnah.o@cmu.ac.th (P.O.); 3 Department of Pharmacology, Faculty of Medicine, Chiang Mai University, Chiang Mai 50200, Thailand; 4 Division of Infectious Diseases, Department of Pediatrics, Faculty of Medicine, Chiang Mai University, Chiang Mai 50200, Thailand; 5 Department of Medicine, Nakornping Hospital, Chiang Mai 50180, Thailand; puntapong@yahoo.com; 6 Department of Pharmacy, Nakornping Hospital, Chiang Mai 50180, Thailand; teera039@gmail.com

**Keywords:** loading dose, colistin, nebulized, CRAB, critically ill patients

## Abstract

Carbapenem-resistant *Acinetobacter baumannii* (CRAB) infections pose a serious threat, with high morbidity and mortality rates. This retrospective cohort study, conducted at Nakornping Hospital between January 2015 and October 2022, aimed to evaluate the efficacy and safety of a high loading dose (LD) of colistin combined with nebulized colistin in critically ill patients with CRAB pneumonia. Of the 261 patients included, 95 received LD colistin, and 166 received LD colistin with nebulized colistin. Multivariate Cox regression analysis, adjusted for baseline covariates using inverse probability weighting, showed no significant difference in 30-day survival between patients who received LD colistin and those who received LD colistin with nebulized colistin (adjusted hazard ratio [aHR]: 1.17, 95% confidence interval [CI]: 0.80–1.72, *p* = 0.418). Likewise, there were no significant differences in clinical response (aHR: 0.93, 95% CI: 0.66–1.31, *p* = 0.688), microbiological response (aHR: 1.21, 95% CI: 0.85–1.73, *p* = 0.279), or nephrotoxicity (aHR: 1.14, 95% CI: 0.79–1.64, *p* = 0.492) between the two treatment groups. No significant adverse events related to nebulized colistin were reported. These findings suggest that the addition of nebulized colistin may not offer additional benefits in terms of 30-day survival, clinical or microbiological response, or nephrotoxicity in these patients.

## 1. Introduction

Carbapenem-resistant *Acinetobacter baumannii* (CRAB) have been associated with serious infections and become an important public health challenge worldwide [1,2]. CRAB infections have shown resistance to a wide range of antimicrobials, including carbapenems, which serve as a model for CRAB or pandrug-resistant (PDR) bacteria. CRAB is a rapidly evolving pathogen, especially in the intensive care setting where it can cause a number of infections, including bacteremia, pneumonia/ventilator-associated pneumonia (VAP), meningitis, urinary tract infection, central venous catheter-related infection, and wound infection [3]. The genetic species of CRAB are frequently related to outbreaks, especially in the intensive care unit (ICU) [3].

Colistin has shown efficacy against *Acinetobacter baumannii* and *Pseudomonas aeruginosa*, including various Gram-negative bacteria, and is used as a salvage therapy for patients with MDR *P. aeruginosa* and *A. baumannii* infections [4]. However, there is still uncertainty regarding the optimal dosing regimens for colistin methanesulfonate (CMS). Additionally, a population pharmacokinetic study of CMS at a conventional dosing schedule of 100 mg of colistin base (3 million units [MU]) every 8 h revealed that colistin has a prolonged half-life, leading to insufficient concentrations during the first 12 to 48 h of treatment initiation [5]. Consequently, there is a need to consider altering the colistin dosing regimen. Administering a loading dose of 9 MU CMS (equivalent to 300 mg of colistin base) followed by a maintenance dose of 4.5 MU CMS (equivalent to 150 mg of colistin base) every 12 h achieves comparable average steady-state concentrations to the conventional dosing schedule while allowing for rapid attainment of therapeutic concentrations and reducing the need for frequent administration [6]. Furthermore, studies have reported inadequate distribution of intravenous colistin concentrations in lung tissue, especially in cases involving significant airway secretions or pulmonary edema, resulting in ineffective antibacterial activity [7]. To address these limitations, inhaled antibiotics, such as nebulized CMS, have been used to minimize nephrotoxicity and enhance the efficacy of intravenous antibiotics [8]. Although nebulized CMS is commonly employed as an adjunctive or alternative therapy, there is limited evidence regarding the clinical efficacy and safety of administering high doses of colistin in combination with nebulized colistin, particularly in specific critically ill patients. Therefore, this study aims to assess the efficacy and safety of a high loading dose (LD) of colistin versus a high LD of colistin in combination with adjunctive nebulized colistin administration for treating critically ill patients with CRAB pneumonia.

## 2. Results

A total of 2547 cases with colistin were recorded in the hospital database. During the study period, 261 patients met the inclusion criteria, of which 95 patients (36.40%) and 166 patients (63.60%) received LD colistin and LD colistin in combination with nebulized colistin, respectively. The mean age ± SD was 65.51 ± 17.29 years, with 152 patients (58.24%) of the total patients being female. The most frequent underlying conditions were hypertension, cardiovascular disease, and chronic renal disease. Table 1 displays the demographic information of the study participants and comparison of LD colistin and LD colistin combined with nebulized colistin.

Table 2 displays the treatment outcomes of Cox regression analysis for critically ill patients receiving LD colistin and LD colistin with nebulized colistin after inverse probability weighting (IPW). The LD colistin plus nebulized colistin group was not associated with a lower 30-day survival rate than the LD colistin group, according to univariate Cox regression analysis (HR: 1.13, 95% CI: 0.78–1.64; *p* = 0.503). Additionally, there was no difference in the clinical response (HR: 0.97, 95% CI: 0.69–1.36; *p* = 0.853), microbiological response (HR: 1.22, 95% CI: 0.87–1.07; *p* = 0.251), or nephrotoxicity between patients who received LD colistin with nebulized colistin and those who received LD colistin (HR: 1.16, 95% CI: 0.81–1.67; *p* = 0.418). The 30-day survival rate (primary outcome) was not significantly associated with patients who received LD colistin compared to those who received LD colistin with nebulized colistin, according to the multivariate Cox proportional hazard model (IPW using the propensity score) (aHR = 1.17, 95% CI: 0.80–1.72; *p* = 0.418). Subgroup analysis of the survival rate in patients with a SOFA score ≥ 2 who received LD colistin with nebulized colistin did not show a significant difference compared to those who received LD colistin alone (adjusted hazard ratio [aHR]: 1.12, 95% CI: 0.71–1.78; *p* = 0.625). Furthermore, LD colistin with nebulized colistin was not associated with clinical response (aHR: 0.93, 95% CI: 0.66–1.31; *p* = 0.688) and microbiological response (aHR: 1.21, 95% CI: 0.85–1.73; *p* = 0.279) compared to LD colistin, according to the secondary outcomes of the IPW propensity score analysis using the Cox regression model. Additionally, there was no difference in the nephrotoxicity rate of LD colistin compared to LD colistin with nebulized colistin (aHR: 1.14, 95% CI: 0.79–1.64; *p* = 0.492) (Table 2).

## 3. Discussion

Our analysis showed that the addition of nebulized colistin did not result in a lower 30-day survival rate when compared to the group receiving LD colistin alone. This finding was supported by both univariate and multivariate Cox regression analyses. Furthermore, no significant difference in clinical response or microbiological response was observed between the two groups. This suggests that the addition of nebulized colistin did not provide additional benefits in terms of resolving symptoms or eradicating the infection compared to LD colistin alone. The efficacy of colistin for the treatment of hospital-acquired pneumonia (HAP) or ventilator-associated pneumonia (VAP) has been a topic of debate due to its limited penetration into the lung tissue. To overcome this limitation, aerosolized colistin has been proposed as an adjunct to intravenous administration to achieve higher concentrations in the lungs [9]. However, the optimal route and dosing of colistin administration remain unclear. There is currently no consensus on the effectiveness of inhaled polymyxin therapy. The guidance provided by the Infectious Diseases Society of America (IDSA) does not recommend the addition of nebulized antibiotics for treating respiratory infections caused by CRAB [10]. According to the panel, the use of nebulized antibiotics as adjunctive therapy for CRAB pneumonia is not recommended due to the lack of observed benefits in clinical trials. Concerns regarding uneven distribution in infected lungs and the potential for respiratory complications, such as bronchoconstriction affecting 10–20% of patients receiving nebulized antibiotics, also contribute to this recommendation [10].

In our study, we investigated the impact of adding nebulized colistin to intravenous (IV) colistin treatment on mortality outcomes in patients with CRAB pneumonia. However, our findings align with a recently published meta-analysis, indicating no statistically significant difference in mortality between the two treatment groups [11,12]. Achieving complete microbiological eradication in critically ill patients can be challenging due to the presence of cross-infection and drug-resistant pathogens. Regarding the outcome of microbiological eradication, our findings were in line with the most recent meta-analysis, which reported no significant difference in microbiological eradication between the two treatment groups [12]. A randomized controlled study conducted at Siriraj Hospital in Thailand investigated the safety and efficacy of nebulized CMS as adjunctive therapy for Gram-negative ventilator-associated pneumonia (VAP). The study included 100 adults with Gram-negative VAP, and the patients were randomized to receive nebulized NSS or nebulized CMS in addition to systemic antibiotic therapy. The results of the study showed that there were no significant differences in the favorable clinical outcome between the nebulized CMS group and the control group (51.0% vs. 53.1%, *p* = 0.84). However, a significantly higher percentage of patients in the nebulized CMS group had a favorable microbiological outcome compared to the control group (60.9% vs. 38.2%, *p* = 0.03). The incidence of bronchospasm was observed in 7.8% of patients in the nebulized CMS group and in 2.0% of patients in the control group (*p* = 0.36). The occurrence of renal impairment was similar between the nebulized CMS group and the NSS group (25.5% vs. 22.4%, *p* = 0.82) [13].

Our study findings are consistent with the Siriraj Hospital study [13], as we also did not observe a significant difference in clinical response or nephrotoxicity between the group receiving nebulized colistin and the group receiving intravenous colistin alone. However, it should be noted that the outcomes may vary due to differences in patient populations, study designs, and treatment protocols. Another important aspect of our analysis was the assessment of nephrotoxicity, a known adverse effect associated with colistin treatment. In the study conducted by Kalin et al., they reported a higher incidence of nephrotoxic events in the IV combination with nebulized colistin group compared to the IV colistin group [14]. However, our results demonstrated no significant difference in the rate of nephrotoxicity between the two treatment groups. This suggests that the addition of nebulized colistin did not increase the risk of renal toxicity when combined with LD colistin. This finding is consistent with previous studies that have reported the safety profile of adjunctive nebulized colistin compared to intravenous colistin, demonstrating no significant increase in the risk of acute kidney injury [15]. The limited systemic absorption of AS colistin, as it primarily exerts its effects locally rather than systemically, may explain the similarity in systemic adverse reactions such as nephrotoxicity between the two treatment groups based on current research evidence.

Moreover, our study evaluated the safety profile of nebulized colistin and found no reported side effects, such as bronchoconstriction, chest tightness, cough, or apnea. This indicates that nebulized colistin was well-tolerated by the patients in our study population. The findings from the multivariate Cox regression analysis demonstrated that the use of LD colistin with nebulized colistin was not associated with a lower 30-day survival rate compared to LD colistin alone. This suggests that the addition of nebulized colistin did not provide a significant survival benefit in patients with CRAB pneumonia. Similarly, there were no significant differences in the clinical and microbiological responses between the two groups. These results indicate that the combination therapy of LD colistin with nebulized colistin did not offer additional clinical or bacteriological benefits over LD colistin alone. The results of our study contribute to the existing knowledge on the optimal treatment strategies for CRAB pneumonia. While previous studies have suggested the potential benefits of nebulized colistin in improving lung concentrations and reducing systemic toxicity, our findings did not support these claims. It is possible that the selected dose and duration of nebulized colistin in our study were not sufficient to demonstrate significant clinical and microbiological benefits. Further investigations on different dosing regimens and larger sample sizes are warranted to confirm our findings.

Several limitations can be noted in our study. Firstly, the study design was retrospective, which may have introduced biases and limited the control over confounding variables. The decision to administer nebulized colistin was not randomized but based on the clinician’s judgment, which could have influenced the outcomes. However, inverse probability weighting (IPW) was employed after adjusting the Cox regression analysis to minimize the influence of confounding factors in our analysis. This approach allowed us to account for potential imbalances in baseline characteristics and reduce the impact of confounders on our results. Secondly, the sample size of our study was relatively small, which may have limited the power to detect small differences between the treatment groups. In addition, the study was a single center in northern Thailand, which may limit the generalizability of our findings to other settings. Thirdly, the retrospective nature of our study led to the limitation of not having a control group that received no colistin treatment. Consequently, we lacked a control group without colistin treatment. Our conclusion regarding the lack of significant differences in outcomes between patients receiving LD colistin with nebulized colistin and those receiving LD colistin alone is based on the comparison within our study population.

## 4. Materials and Methods

### 4.1. Study Design and Population

A retrospective cohort study was conducted at Nakornping Hospital, an 800-bed tertiary and regional hospital in Chiang Mai. The study participants’ details were extracted from January 2015 to October 2022. The study utilized the definitions of pneumonia established by the Centers for Disease Control and Prevention (CDC), which refer to an infection of the lungs which can result in mild to severe infection [16]. The inclusion criteria consisted of critically ill patients who were 18 years of age or older and had received either a high LD of colistin or a combination of LD colistin with nebulized colistin for the treatment of documented CRAB pneumonia for more than 72 h. Additionally, the patients included in the study were admitted to ICU. Exclusion criteria included patients who received renal replacement therapy, were pregnant or breastfeeding, were receiving concomitant antibiotics effective against CRAB, or had baseline end-stage renal disease resulting from acute kidney injury (AKI). Patients with contamination or colonization in their CRAB cultures, as well as those with insufficient electronic medical record documentation, were also excluded from the study. The research protocol was approved by the ethics committee on human research at Nakornping Hospital (093/65). The patients who received LD colistin and those who received LD colistin with nebulized colistin were divided into two distinct groups for analysis purposes.

### 4.2. Sample Size

A sample size of 144 patients was determined based on the 28-day all-cause mortality of nebulized colistin in combination with intravenous antibiotics, with a hazard ratio (HR) of 0.499 [17]. To achieve the required number of events with an accepted 5% type I error and 20% type II error, 72 patients were needed in each group [17,18].

### 4.3. Colistin Administration

The formulation of CMS in Thailand is 4.5 million units per vial, which is equivalent to 150 mg of colistin base activity (CBA). Colistin was administered by dilution in 100 mL of normal saline and was given over 1 h. The treatment protocol consisted of an initial loading dose of 300 mg of colistin base, followed by a maintenance dose of 150 mg of colistin base every 12 h. Additionally, aerosolized colistin was administered immediately upon reconstitution via either a jet or ultrasonic nebulizer. The dosage was equivalent to 75 mg of colistin base in normal saline (NSS) and was given daily, every 8 h, until completion of systemic colistin therapy for pneumonia. This approach ensured uniformity in drug delivery among all participants.

### 4.4. Data Collection

Patients with CRAB infections were identified based on the microbiology laboratory data and hospital number (HNs). The data collected included patient demographics, clinical diagnosis, underlying diseases, duration of colistin therapy, culture data, source of infection (as documented in the medical record by the treating physicians), daily colistin dose, length of stay until start of colistin, concomitant antibiotic therapy dose and duration, timing of antibiotic therapy, mortality and Sequential Organ Failure Assessment (SOFA) scores and APACHE II score obtained on the day of admission to the hospital. Patient data were captured in case record forms (CRFs) and verified before data cleaning and statistical analysis. Patient information was anonymous and treated as confidential using the study ID throughout the data collection process.

### 4.5. Definition

Pneumonia was identified according to specific diagnostic criteria. These criteria included the presence of a new and progressive pulmonary infiltrate observed on radiographic imaging, along with at least two of the following clinical indicators: body temperature exceeding 38 °C or falling below 35.5 °C, leukocytosis (leukocyte count greater than 12,000 cells/mm^3^) or leukopenia (leukocyte count less than 4000 cells/mm^3^), and clinical evidence suggesting pneumonia, such as the presence of purulent bronchial secretions and a decrease in oxygenation [16].

### 4.6. Efficacy Outcome

The evaluation of efficacy was based on the 30-day survival rate, as well as the clinical and bacteriological responses observed after 30 days of colistin therapy. The primary outcome measure focused on the survival rate after 30 days. Secondary outcomes included the assessed clinical and bacteriological outcomes of pneumonia infection and any adverse events associated with colistin treatment.

The clinical response of pneumonia was defined based on various criteria, including clinical, radiological, and laboratory findings. A favorable clinical response (cure or improvement) was determined by the resolution or partial resolution of presenting symptoms and signs of pneumonia, a decrease in suctioning requirements, improvement in radiographic findings on chest X-ray, and normalization of laboratory parameters such as arterial blood gases and white blood cell count [19].

On the other hand, clinical failure (unresponsiveness) was defined as the persistence or worsening of presenting symptoms and/or signs of infection during the administration of colistin. Bacteriological clearance referred to the eradication of CRAB isolates as confirmed by follow-up cultures. Conversely, bacteriological failure indicated the persistence of CRAB isolates on follow-up culture, suggesting an incomplete eradication of the infection.

### 4.7. Safety Outcomes

Safety was evaluated based on changes from baseline in serum urea and creatinine levels following colistin therapy. These results were obtained from laboratory tests for renal function. The RIFLE criteria, which distinguish 5 categories of renal insufficiency, risk, injury, failure, loss, and end-stage, were used to compare renal toxicity between the beginning and completion of treatment.

### 4.8. Statistical Analysis

Data analysis was performed using Stata software, version 14 (Stata Corp, College Station, TX, USA). The comparison of colistin treatment was conducted between the two treatment groups (a high LD colistin vs. a high LD colistin in combination with adjunctive nebulized colistin). The list-wise deletion method was employed to handle missing values. In this method, an entire record or observation would be removed if it contained any missing values [20]. Descriptive statistics, including percentages, frequencies, means, and standard deviations, were used to summarize the patients’ general characteristics and fundamental information, enabling a primary understanding of the study outcomes. For categorical variables, the Chi-square test or Fisher’s exact test was applied, while continuous variables were assessed using *t*-tests or Mann–Whitney U tests, as appropriate. When the data followed a normal distribution, independent *t*-tests were used to determine the statistical differences in means across various methods. Conversely, when the data were irregularly or non-normally distributed, the Mann–Whitney U test was utilized. A two-tailed *p*-value of less than 0.05 was considered statistically significant.

Due to imbalances in the baseline characteristics of the treatment groups, inverse probability weighting (IPW) propensity score modification was carried out to reduce potential biases. The application of probability weights in the inverse probability of treatment weighting contributes to balancing out the disparity in potential confounding factors between treated and control patients. We calculated a propensity score using multivariable logistic regression. The variables used to calculate the propensity score included baseline covariates with an inclusion criterion of *p*-value < 0.25 [21] (age, APACHE II score, hypertension, baseline GFR, and aminoglycosides) and variables that were likely to influence the outcomes (septic shock). The outcomes for the two therapy groups were then examined using the weights, taking into account the time-to-event nature of the data with cause-specific Cox proportional hazard regression models.

A univariate Cox regression analysis was used to investigate variables that were associated with both the primary (30-day survival) and secondary (i.e., clinical response, microbiological response, and nephrotoxicity) outcomes. Inverse probability weighting employing the propensity score for baseline covariate adjustment was utilized to analyze a multiple-variable analysis and obtain the adjusted hazard ratios (HRs) and 95% confidence intervals (CIs) of relevant components. A 2-sided = 0.05 was recognized as statistically significant for all analyses.

### 4.9. Antimicrobial Susceptibility Testing

*A. baumannii* isolates were identified at the Clinical Microbiology division of Nakornping Hospital using conventional culture techniques and biochemical methods. Standard microbiological approaches were employed to identify all pathogenic bacteria in the study. Both the automated broth microdilution method (VITEK 2 system, bioMérieux, Durham, NC, USA) and the disk diffusion method were used for the susceptibility testing. According to the Clinical and Laboratory Standards Institute (CLSI) methodology, antimicrobial susceptibility was assessed [22]. The VITEK 2 system was used to test *A. baumannii*’s susceptibility to antibiotics, and broth microdilution was used to test its susceptibility to colistin. Resistance was defined as having a colistin minimum inhibitory concentration (MIC) breakpoint greater than 2 mg/L. The VITEK 2 system is a completely automated system that uses a turbidimetric method for susceptibility testing and a fluorogenic methodology for identifying organisms [23]. *A. baumannii,* which is susceptible to colistin but resistant to carbapenems, is identified as CRAB.

## 5. Conclusions

In conclusion, our study did not find significant differences in the 30-day survival rate, clinical response, microbiological response, or nephrotoxicity between patients who received LD colistin with nebulized colistin and those who received LD colistin alone for the treatment of CRAB pneumonia. These findings suggest that the addition of nebulized colistin may not provide additional benefits in terms of efficacy and safety compared to LD colistin alone. Further prospective studies with larger sample sizes and different dosing regimens are needed to validate these findings and provide more robust evidence on the optimal treatment approach for CRAB pneumonia.

## Figures and Tables

**Table 1:** Demographic and clinical characteristics of patients receiving LD colistin compared to LD colistin with nebulized colistin therapy.

Characteristic	LD Colistin (n = 95)	LD Colistin with Nebulized Colistin (n = 166)	*p*-Value
Sex, n (%)			
Male	44 (46.32)	65 (39.16)	0.259
Female	51 (53.68)	101 (60.84)	
Age, mean ± SD (year)	61.53 ± 18.76	67.78 ± 16.01	0.005 *
Septic shock, n (%)	68 (71.58)	124 (74.70)	0.582 *
Norepinephrine	61 (64.21)	109 (65.66)	0.813
Dopamine	6 (6.32)	17 (10.24)	0.282
Mechanical ventilation, n (%)	89 (93.68)	156 (93.98)	0.925
Duration of treatment, mean ± SD (day)	9.51 ± 5.33	10.16 ± 7.63	0.462
APACHE II score, mean ± SD	17.19 ± 4.27	18.18 ± 4.64	0.089 *
SOFA score, mean ± SD	2.87 ± 0.18	2.89 ± 0.14	0.319
Baseline SCr, mean ± SD (mg/dL)	1.39 ± 1.65	1.42 ± 1.20	0.879
Baseline GFR, mean ± SD (mL/min)	65.74 ± 47.84	49.77 ± 44.25	0.008 *
Comorbidities * n (%)			
Hypertension	40 (42.11)	85 (51.20)	0.157 *
Cardiovascular disease	35 (36.84)	55 (33.13)	0.544
Diabetes mellitus	18 (18.95)	37 (22.29)	0.524
Chronic kidney disease	21 (22.11)	40 (24.10)	0.715
Chronic obstructive pulmonary disease	18 (18.95)	38 (22.89)	0.455
Malignancy	18 (18.95)	37 (22.29)	0.524
Chronic liver disease	6 (6.32)	9 (5.42)	0.765
Previous surgery	7 (7.37)	11 (6.63)	0.820
Type of nephrotoxic medications ^#^, n (%)			
Aminoglycosides	4 (4.21)	2 (1.20)	0.119 *
Diuretics	82 (86.32)	141 (84.94)	0.762
Amphotericin B	11 (11.58)	12 (7.23)	0.233
Vasopressor	67 (70.53)	126 (75.90)	0.341
Vancomycin	59 (62.11)	113 (68.07)	0.328
Colistin MIC, median (min–max)	0.25 (0.094–1.5)	0.25 (0.064–1.5)	0.898

SCr, serum creatinine; SD, standard deviation; LD, loading dose; APACHE II, acute physiology and chronic health evaluation II; SOFA, sequential organ failure assessment; GFR, glomerular filtration rate; MIC, minimal inhibitory concentration; Other, inter-costal drainage and pus from wound. * Used for adjusting confounding factors. ^#^ Each patient could have more than 1 drug.

**Table 2:** Cox regression analysis of outcomes for critically ill patients receiving LD colistin and LD colistin with nebulized colistin after inverse probability weighting (IPW) (n = 261).

Variable	LD Colistin Monotherapy (n = 95)	LD Colistin with Nebulized Colistin (n = 166)	Crude HR (95% CI)	*p*-Value	Adjusted HR * (95% CI)	*p*-Value
Efficacy Primary outcomes						
30-day survival	47 (49.7)	69 (41.6)	1.13 (0.78–1.64)	0.503	1.17 (0.80–1.72)	0.418
Survival in SOFA score ≥ 2	19 (20.00)	31 (18.67)	1.08 (0.69–1.70)	0.737	1.12 (0.71–1.78)	0.625
Secondary outcomes						
Clinical response	52 (55.2)	90 (54.1)	0.97 (0.69–1.36)	0.853	0.93 (0.66–1.31)	0.688
Microbiological response	63 (66.5)	86 (52.1)	1.22 (0.87–1.70)	0.251	1.21 (0.85–1.73)	0.279
Safety						
Nephrotoxicity (RIFLE criteria)	53 (55.6)	73 (44.1)	1.16 (0.81–1.67)	0.418	1.14 (0.79–1.64)	0.492
Risk	21 (39.7)	37 (41.7)				
Injury	12 (22.8)	17 (23.6)				
Failure	11 (20.6)	11 (14.4)				
Loss	9 (16.2)	13 (17.7)				
ESRD	0 (0.0)	2 (1.3)				

LD, loading dose; CI, confidence interval; HR, hazard ratio; * Adjusted using inverse probability weighting (IPW) with the propensity score for baseline covariate adjustment.

## Data Availability

The datasets used and analyzed during the current study are available from the corresponding author on reasonable request.
